# Transformations through Proximity Flying: A Phenomenological Investigation

**DOI:** 10.3389/fpsyg.2017.01831

**Published:** 2017-10-17

**Authors:** Maria Holmbom, Eric Brymer, Robert D. Schweitzer

**Affiliations:** ^1^Department of Psychology, Umeå University, Umeå, Sweden; ^2^Institute of Sport, Physical Activity and Leisure, Leeds Beckett University, Leeds, United Kingdom; ^3^School of Psychology and Counselling, Queensland University of Technology, Brisbane, QLD, Australia

**Keywords:** extreme sports, proximity flying, personal transformation, phenomenology, well-being, identity

## Abstract

Participation in extreme sports has been linked to personal transformations in everyday life. Descriptions of lived experience resulting from transformative experiences are limited. Proximity flying, a relatively new discipline involving BASE jumping with a wingsuit where participants fly close to solid structures, is arguably one of the most extreme of extreme sports. The aim of this paper, part of a larger phenomenological study on the lived experience of proximity flying, is to explicate the ways in which participating in proximity flying influences the everyday lives of participants. Interpretative phenomenological analysis was used to explicate the lived experience of six proximity pilots. An analysis of interview transcripts revealed three significant themes describing the lived experience of participants. First, experiences of change were described as positive and skills developed through proximity flying were transferable into everyday life. Second, transformative experiences were considered fundamental to participants’ perspectives on life. Third, experience of transformation influenced their sense of personal identity and facilitated flourishing in other aspects of everyday life. Participants were clear that their experiences in proximity flying facilitated a profound process of transformation which manifest as changes in everyday capabilities and behaviors, values and sense of identity.

## Introduction

Over the last 40 years research on extreme sport participants has focused on providing explanations for participants engaging in a leisure activity, where death is the most likely consequence of a mismanaged mistake or accident (Brymer and Schweitzer, 2017). The dominant, theory-driven perspective on extreme sport participation focusses upon understanding personality and motivations placing extreme sport athletes in a similar category to illicit drug takers and deviant social groups. That is, there is an argument that motivation for engaging in such activities stems from deep seated personality deficits that manifest as a basic, pathological need for thrills and risk (e.g., Zuckerman, 1994; Elmes and Barry, 1999; Franques et al., 2003; Pain and Pain, 2005; Self et al., 2007). From this perspective, extreme sport participants are typically considered selfish, young males ‘fascinated with the individuality, risk, and danger of the sports’ (Bennett et al., 2003, p. 98). Extreme sport participation is most often explained as the need for an adrenaline rush or because participants are just plain crazy trying to prove themselves worthy of respect by fighting nature or taking unnecessary risks (Le Breton, 2000; Monasterio, 2007).

The traditional risk focus that presupposes that extreme sports are about undesirable risk taking is being questioned as more recent studies in personality and psychology report that while risk and deviancy might explain the motives of some participants, the variance is too great to be useful to describe a participant population more generally (Woodman et al., 2010; Kerr and Houge Mackenzie, 2012; Monasterio et al., 2014). Furthermore, there is an emerging body of research, often drawing upon phenomenology, challenging traditional assumptions, and presenting more nuanced perspectives on motivations for participation in extreme sports (Willig, 2008; Brymer and Gray, 2009; Brymer and Oades, 2009; Brymer et al., 2009; Kerr and Houge Mackenzie, 2012; Brymer and Schweitzer, 2013, 2014; Simpson et al., 2014; Wiersma, 2014). Results from these studies suggest that while risk and thrills might underlie initial motivations for some participants, risk-seeking is not reflected in the deliberations of more experienced athletes who invariably refute these assertions (Celsi et al., 1993; Willig, 2008; Brymer and Oades, 2009; Kerr and Houge Mackenzie, 2012). Experienced extreme sport athletes are more likely to associate participation with positive and life enhancing experiences, either because the act itself (e.g., catching and surfing a big wave) is meaningful or due to the fact that the extreme sport experience is itself transformational (Brymer, 2013). While a number of studies have documented the experience of participation, the transformational hypothesis is less well developed (Brymer, 2013). In this paper, we present results from a larger phenomenological study with six highly experienced extreme sport athletes who engage in proximity flying, outlining their everyday lived experience which they report has been transformed as a result of proximity flying.

### Transformations through Extreme Sports

In recent years, research has indicated that extreme sports are associated with positive life enhancing change. Researchers point to the potential for extreme sports to transform everyday lived experience (Willig, 2008). However, descriptions of the outcomes of being transformed are limited. Extreme sports have been promoted as a medium for learning about goal achievement and mastery (Willig, 2008; Allman et al., 2009; Kerr and Houge Mackenzie, 2012), overcoming and managing fear and anxiety (Allman et al., 2009; Kerr and Houge Mackenzie, 2012; Brymer and Schweitzer, 2013), developing self-efficacy (Slanger and Rudestam, 1997), and increasing self-esteem (Willig, 2008). These attributes have the potential to enhance participants’ sense of self, and quality of life.

Extreme sports have also been associated with the development of a new sense of identity (Celsi et al., 1993; Maeland, 2002, unpublished; Larkin and Griffiths, 2004; Allman et al., 2009; Brymer and Oades, 2009). For example, Brymer (2013) argued that extreme sports facilitated ‘an inner journey to self-discovery and an opportunity for reflecting on personal capabilities’ (p. 115). Participation in extreme sports has been posited as providing opportunities to transcend limited ideas of ‘self’ and discovering a newly found and fulfilling sense of identity coupled with corresponding capabilities (Willig, 2008). However, neither Willig (2008) nor Brymer (2013) documented in what ways this might manifest in everyday life. Brymer and Schweitzer (2013) argued that the experience of fear inherent in extreme sports is embraced by athletes and used positively not just in order to ensure effective participation but also for overall psychological health and wellbeing. There was no attempt to describe the lived experience of extreme sport participants’ renewed sense of wellbeing. Despite the lack of evidence or descriptions on how everyday life is lived, the notion that extreme sports transform everyday life in profound and positive ways has attracted some research attention (Willig, 2008; Allman et al., 2009; Brymer and Oades, 2009; Simpson et al., 2014). For some researchers, the notion has prompted claims that athletes consciously and deliberately utilize extreme sports in order to experience positive transformation (Allman et al., 2009). This article, part of a larger phenomenological study on proximity flying, articulates the everyday lived experience of pilots who engage in proximity flying. Findings indicate a direct link between participation in extreme sports and changes in the ways that everyday life is lived thus providing a tantalizing glimpse into how extreme sports might positively influence the everyday lives of participants.

#### Proximity Flying

Proximity flying is one of the most extreme of extreme sports, and arguably unique in guiding the pilot within a space between exhilaration and death, like no other sport experience. Proximity flying is a relatively new addition to BASE jumping (Mei-Dan et al., 2013). BASE is an acronym that stands for the four most common categories of objects from which an individual may jump: building, antenna, span, and earth (cliffs). The participants in proximity flying wear an inflatable ram-air wing design suit, called a wingsuit, which adds surface area to the body and allows forward motion. The airfoil design means that the pilots can glide horizontally for approximately 2–2.5 m for every meter they fall vertically (Berry et al., 2010). Like traditional BASE jumping, proximity pilots jump from solid, usually natural structures such as cliffs and mountains. Take off, otherwise known as launching, where the participant launches from the solid structure into space, is crucial in proximity flying since the participant needs to achieve particular body orientation for flight in a context of limited speed and maneuverability (Mei-Dan et al., 2013).

In the early days of BASE jumping with a wingsuit, the wingsuit design was smaller and pilots flew away from the cliffs to achieve a safe distance. The improved technology and the increased glide ratio of more modern designs allows ‘flying’ in close proximity to the ground, trees, and ridges either in brief flybys or extended terrain flying (Mei-Dan et al., 2013). This evolution in wingsuit BASE jumping is called proximity flying. Proximity pilots, like traditional BASE jumpers, use only one parachute compared to skydivers’ two, and the parachute is deployed closer to the ground than in skydiving, often less than 150 m (altitude 500 ft) (Westman et al., 2008). Mei-Dan et al. (2013) reported that in 2013, approximately 90% of the deaths in BASE jumping were related to the use of wingsuits. The most common fatal mistake in proximity flying is glide path miscalculation and/or limited experience, resulting in cliff or ground impact (Mei-Dan et al., 2013). Proximity flyers are thus encouraged to undertake rigorous training in BASE jumping and wingsuit skydiving, with a suggested minimum of 500 BASE jumps and to continuously practice emergency procedures on the ground, all as prerequisite to flying. Skydiving is a common training arena for wingsuit flying techniques as launching from airplanes means the pilot can avoid physical obstacles inherent in proximity flying.

### Confronting Death, Transformation, and Authenticity

Theoretical perspectives from existential psychology suggest that accepting personal vulnerabilities, facing death, and experiencing the potential reality of our own death leads to a process of transformation encompassing fundamental values in the ways in which we experience our sense of self. Change in this context is understood in terms of a process of transformation resulting in a life lived more fully and with greater authenticity. The term transformation is seen as particularly apt, in that it is derived from the Latin, *transformatio*, referring to “change of shape.” The shape of one’s life is thus changed in that the experience leads to a life lived fully and authentically, where authenticity is understood as taking responsibility for one’s own existence rather than following the crowd even though this might create discomfort (May, 1983). Being authentic is not an isolated end point but experienced as a process of becoming more true to oneself and accepting limitations and possibilities.

Rogers (1961) expressed this concept in terms of living one’s life according to the needs of one’s inner being, rather than the demands of society or early conditioning. Values and identities are not constructed from scratch but particular events or activities allow us to focus on the reality of our own finitude and facilitate a realization of our potential and our sense of who we are (Park, 2006). Life takes on a new, profound and positive meaning (Wong, 1998, 2000; Emmons, 1999). The end result of confronting and accepting the reality of our own death without self-importance is that both the sense of who we are, and our relationship with others are enhanced (Wong, 2000). From an existential perspective recognizing physical limitations, such as the temporary nature of physical security, through accepting the reality of our own death releases a capacity to live a full and meaningful life.

Heidegger recognized this notion as a paradox, where an experiential acceptance of the reality of our own death reveals the extent to which we have developed protective circumventions in our own life in order to protect ourselves against the challenge of living authentically. Revealing these protective strategies can facilitate a process of becoming more free, integrated, focused and centered, and thereby support learning to live more authentic lives (Zimmerman, 1986, p. 292). Extreme sports, providing participants have the appropriate personal characteristics (e.g., resiliency, optimism, and hardiness), can be described as extraordinary events that seem to facilitate increasing self-awareness leading to experiences of transformation in the way a participant lives her or his everyday life (Brymer and Oades, 2009; Brymer, 2013).

The notion of transformation as articulated above is reflected in both the literature on transformative learning and in reference to eudaimonia. Transformative learning is conceptualized as “processes that result in significant and irreversible changes in the way a person experiences, conceptualizes, and interacts with the world” (Hoggan, 2016, p. 71). Learning can happen instantaneously and manifests as profound shifts in consciousness and the ways in which we understand ourselves, the natural world, and others (Brymer, 2013). Eudaimonia has its origin in Greek, with “*eu*” (good) and “*daimon*” (“spirit”). The term thus refers to the realization of one’s potential, and calls upon us to fulfill our destiny by recognizing and living with one’s daimon, or “true self” (Norton, 1976). Eudaimonia may thus refer to the feelings available when one is moving toward self-realization and furthering one’s purpose in living through activity. Certain events, such as those that bring us closer to realizing and accepting the inevitability of our own death, provide a medium for momentous realizations which ‘energizes us to live with vitality and purpose’ (Wong, 2008, p. 69). This realization of a deeper sense of potential facilitates a clearer appreciation of personal needs, wants and desires (Wong, 2008). According to Wong and Tomer (2011) a positive acceptance of the reality of our own death, as outlined by meaning management theory (MMT) rather than the management of terror assumed to be linked to experiences of death, predicts a life lived by ‘maximizing meaning, fulfillment and joy’ (Wong and Tomer, 2011, p. 104). That is, the transformations experienced in these instances are life enhancing and result from being connected to, or more in tune with a deeper sense of self which has been hidden from the person’s view ‘by socio-cultural noise and interference; noise that dictates who we ‘should’ be and how we ‘should’ lead life’ (Brymer, 2013, p. 6). Arguably, the extreme sport experience acts as a facilitator that removes barriers to noticing the noise and interference and presents an opportunity for an individual to hear their authentic ‘own-self.’ In Heideggerian terms ‘in order to gain everything, one must give up everything’ (Zimmerman, 1986, p. 292).

## Materials and Methods

Qualitative methodologies allow for the rich description of novel human experiences, with particular salience in exploring the lived experience of participants. The current study draws from a larger phenomenological study seeking to explicate the experiences of elite wingsuit pilots’ engaging in proximity flying. Ethical approval for the larger study was in line with the requirements of the University of the first author. Interpretive Phenomenological Analysis (IPA) provides both an inductive and systematized qualitative methodology to address the aims of the study.

Interpretive Phenomenological Analysis draws on key features from phenomenology, hermeneutics, and ideography. Within the field of psychology, phenomenology focuses upon phenomena as experienced. Phenomenology draws upon the philosophical principles originally developed by Husserl (1859–1938) who wished to find an absolute basis for knowing or knowledge. Husserl challenged what has come to be called “scientism” and sought to return to fundamental human experience, which he believed may be understood in terms of intentionality (Brymer and Schweitzer, 2017). From this perspective, the researcher attempts to capture the nature, the quality, and the meaning of participant experience. To understand the lived experience of phenomena, the researcher adopts a phenomenological paradigm. In contrast to the dominant natural science paradigm which privileges objectivity phenomenology seeks to gain understanding by processing data in an iterative manner referred to as a hermeneutic circle. That is, a methodological process of understanding the whole by reference to the parts, and at the same time, understanding parts by reference to the whole. Hermeneutics is more commonly applied to the understanding of texts, but may also play a role in a psychological methodology where the aim is to understand human experience and the meanings attributed to experience (Willig, 2013). IPA draws upon in-depth accounts produced by participants, for example diary notes or transcribed interview data (Willig, 2013). In the larger study, in-depth interviews were used to reveal participants’ ways of understanding their personal experience of proximity flying. IPA is idiographic through encouraging an in-depth study of individual cases, focusing on particular individuals, but also nomothetic, as the findings are integrated and represent the experience of the group (Smith et al., 2009).

Crucial in phenomenology is for the researcher to become aware of personal presuppositions and assumptions about phenomena and consciously put them aside, which is referred to as bracketing, with a view to being open to the phenomenon as it presents itself in all its particularity. As part of the process of bracketing the first author created a mind-map representing personal and theoretical presuppositions before conducting the study in order to make assumptions explicit and facilitate reflections on potential bias in interviewing and explicating the data. The mind-map was continually referred to and updated. In the initial stages this process facilitated self-reflection about undertaking the study and the development of appropriate questions. The mind-map which operationalized the process of bracketing was used as a personal tool throughout the writing process to remind the researchers of possible bias. This resulted in a strong awareness of values, perceptions, expectations and feelings about extreme sports and proximity flying, e.g., that the wingsuit pilots mostly are intrinsically motivated. The second and third author also carried out a reflexive process which made explicit their own biases as part of the data analysis dialog. In the following sections, we outline how the study was undertaken.

### Participants

Participants were recruited by the first author following purposive sampling. To be included in the project pilots needed to be highly experienced in proximity flying and willing to explore the proximity flying experience. Participants also needed to be able to communicate their experience in English or in Swedish. Twenty-seven (25 males and 2 females) proximity pilots who competed in either the 2013 World Wingsuit League in the Tianmen Mountains, China, or the 2013 HeliBASE 74, in Switzerland were initially contacted. Two male pilots experienced fatal accidents while proximity flying in 2013. Eight pilots who met the criteria accepted the invitation and six male proximity wingsuit pilots eventually participated in the study. Two pilots were unable to meet for interviews either online or in person. Participants were over 30 years of age and heralded from Europe, Australasia, and the Americas. Five of the participants were married; one had children. All participants were either sponsored or professional extreme sport athletes.

### Interviews

Following the provision of informed consent, interviews were conducted by the first author using a semi-structured approach through online video conversations. Interviews were recorded for later transcription. The first interview was conducted in Swedish and the following five interviews were conducted in English. All interviews were guided by the research question ‘what is your experience of proximity flying’? Specifically, pilots were asked; (1) *Please share your stories about how you discovered proximity flying* and (2) *Please describe a particular proximity flight with as much detail as possible*. In order to facilitate deep reflection and enhance the potential of rich description these two questions were sent by e-mail to participants prior to the interviews. To gain a more in-depth understanding of the experience of proximity flying, the interviewer followed up with contextualized prompts, such as: ‘Please tell me more’ or ‘please could you expand’? Emphasis was placed upon specific experiences with the intention of eliciting states of mind, otherwise understood as lived experience as opposed to a chronicle of events. In keeping with the principles of phenomenology, the aim was to elicit a detailed and penetrating description of the experience, with a focus on the meaning and significance of the experience.

### Data Analysis

The interviews were transcribed verbatim and pseudonyms were allocated to each interview transcript to safeguard confidentiality. In the first stage of analysis, the first author listened to each transcript followed by transcribing the audiotape. This was followed by a process of reading and re-reading the transcripts to gain a sense of the data as a whole, focusing upon the participant’s perspective and experiences. In this sense, the investigator continued to adopt a phenomenological stance, in that assumptions were “bracketed,” allowing the data, as far as was possible, to speak for itself. The phenomenological attitude also requires a particular stance based upon an understanding of intentionality. That is, an appreciation that consciousness is not based upon the dominant paradigm of a subject–object view of events, but as consciousness and the object of consciousness, in this instance, the experience of proximity flying, being co-constituted (Brymer and Schweitzer, 2017). Adopting this attitude, the developed texts were shared with the second and third authors in order to facilitate future dialog and the development of the thematic structures.

The second stage involved the first author making initial notes on each transcript focusing on descriptive, linguistic, and conceptual comments which were used to underpin the emerging themes. Descriptive comments were then used to initiate the development of phenomenological themes. Linguistic comments explored the participants’ use of language, for example ‘unable to explain the feeling in words,’ or ‘emphasizing importance by swearing,’ while conceptual comments focused on a more theoretical and questioning form, such as ‘transference of lessons learned’ or ‘a fundamental change?’ Conceptual comments emerging from this process were guided by participants’ reported experience. This stage of analysis was repeated several times for each interview.

The third stage of analysis comprised identifying and labeling emerging themes, focusing on the nature, quality and meaning of participants’ experiences of proximity flying. This stage involved staying close to the participants’ lived-experience. The emerging themes were coded with terms such as ‘transferring skills to everyday life,’ ‘positive mood change,’ ‘life-changing experience,’ and ‘new-found values in life.’ The process of explication was repeated for each transcript. The second and third author provided feedback and facilitated a process whereby emerging constructs were interrogated in terms of their relevance, cross over or connection with other themes.

The fourth stage involved themes being clustered to better portray the finer distinctions expressed by participants. In the fifth stage, the clusters of themes were abstracted into overarching themes. This process was guided by reflective questions: ‘How is this relevant to the phenomenology of proximity flying?’, ‘What is the nature, quality, and meaning in this account?’ (Wiersma, 2014), and ‘What may we be missing?’ All authors contributed to stages four and five, and arrived at a consensus for the development of and explication of the final themes constituting the phenomenon. Themes were referred to participants for comments and checking that they were consistent with the respondents’ lived-experience. Responses received affirmed that the findings reflected participants’ lived experience.

## Results

This article focuses on one important dimension of proximity pilots’ experience, that is, the lived-experience of transformation gained through the extreme sport of proximity flying. The notion of transformation is distinguished from a natural science perspective, of changes conceived as a measured state of difference between before and after, but refers to the recalled experience of participants, and is better understood in terms of lived-experience of how the change is manifest in the everyday consciousness of participants, from the Latin *transformatio*, which refers to “change in shape or metamorphose.” The experiences described by participants reflect the experience of change or “metamorphosis” which is experienced in terms of the discovering of capabilities, values, and the realization of a sense of identity that may have been available to the participant but was, in a phenomenological sense, previously hidden from view.

Three themes emerged from the data revealing changes in everyday capabilities, behaviors, values, and sense of identity (see **Table 1**). Participants clearly recognized and gave voice to the experience of change and that these changes were directly associated with their experience of participating in proximity flying. First, participants reported that these experiences were positive and experienced as manifest in the ways that everyday life was lived. Second, these transformative experiences were considered to be fundamental to participant perspectives on life. Third, these changes, termed transformations influenced their sense of personal identity and facilitated flourishing in other aspects of everyday life. In the following sections we explicate the three overarching themes that emerged as describing the ways in which participation in proximity flying transformed everyday live experience: transferability of capabilities, life as meaningful, and emergence of selfhood. Each of these overarching themes were regarded as aspects of transformation. In order to properly associate these experiences with proximity flying we begin by reporting on the connections made by proximity flyers between participation in their sport and the experience of transformation.

**Table 1:** The clusters of themes and identification of the overarching themes associated with the experience of having been transformed though proximity flying.

Clusters of themes	Overarching themes
Fear management, self-awareness, understanding of others, and the lived world	Transferability of capabilities
Acceptance of mortality facilitates a new perspective on life, embracing life, mindful appreciation	Life as meaningful
Unique identity, breaking free from constraints, authenticity	Emergence of selfhood

Participants were unanimous that participation in proximity flying had transformed them in profound and positive ways. For example, Adam determined that proximity flying profoundly added to his everyday life experiences.

It means so much more to me than the flight in itself. It defines me as a human being, it gives me confidence in many other places in society. It makes me happier, it gives me a lot more energy. It makes me feel that no other challenge is too big. It has such a synergic effect on me as a human being that means incredibly much to me. (Adam)

Here, Adam describes flying as a defining concept that provides a foundation for many positive transformations such as increased confidence, happiness, enhanced energy, and a realization that challenges are never too big to resolve. Eric also described how proximity flying changed him for the better:

When you talk to people who knew me before and know me now, and they’re like: “Dude!” And then you realize that my life has changed … That I am so happy and so complete. That I am more of a centered person, an inspirational person. The sport has brought the best out of me. (Eric)

In the quote above Eric describes how proximity flying was responsible for positive changes in the way he experiences everyday life manifest not just as increased happiness but as a sense of completeness not felt before taking up proximity flying. Participation in proximity flying has facilitated new ways of experiencing life and being in the world.

Once I met this sport, I found an outlet, somewhere to let go of all of my energy. And everything made sense, you know, in life … Some of the things that I used to hold value to in the past, were not important anymore. (Eric)

This notion that proximity flying is responsible for enabling a pilot to tune into fundamental values is recognized by others. For some such as Eric above it is described as an experience of completion and for others about instigating profound self-knowledge. For example, Adam noted that: “the sport, flying, has taught me to know myself” (Adam). In essence all participants reported that extreme sports were responsible for profound and positive changes, not only in terms of behavior and positive psychological outcomes but also in terms of changes in values and identity.

### Transferability of Capabilities

According to participants in this study, the skills required to fly safely and effectively in proximity flying flow over into everyday life. For some this was a direct transfer of skills such as the skills required to manage emotions and the practicalities of emergencies, for others flying provided a new perspective and insights on how to manage everyday life problems and emotions. For example, Adam determined that the skills honed through flying can be used ‘to prepare myself for doing a lecture,’ or even to guide his interpretations of what others might be feeling when being challenged.

It is hard work to eliminate human mistakes and to perfect myself; my body, my head, and my mind, to an immense task. And the work that I do to fly, I can copy that to other tasks in life. It can be to prepare myself for doing a lecture, or using the ability to maybe understand another human being; to see when they might be on their edge, on their exit point, ready to jump. Well, metaphorically… Or if it’s driving or being in a meeting, or going on a date. The sport has made me see other people better… I feel that I am better at understanding when other people have a hard time … I can see if they need a supporting hand or a talk or even a kick in the butt. ^∗^laughter^∗^ (Adam)

In the same vein, Eric and Chase related how the skills required to harness and work through fear in flying were the same ones used in everyday life. Learning how to manage fear teaches that all challenges are surmountable and provides a medium to flourish. Chase described how planning required for successful flying supports the ability to simplify challenges to reveal more easily managed chunks. Chase exemplified this through his description of a typical situation where the transfer is direct and most obvious:

If there is an accident, I can very calmly deal with the situation and then, afterward, you can deal with your emotions and all that. But during that period of time: You go in and you’re calculated. (Chase)

The skills required to fly effectively transfer into everyday life in many ways. For example, flying comes with intense emotions such as fear. Managing the effects of fear in flying is essential for effective participation, however, as Chase informs us in the quote above the skills learnt through proximity flying are not confined to the extreme sport context, they are also transferable into everyday life. Participants noted that experiencing human flight helps them to gain a new perspective on and develop effective skills for everyday events. The skills required to break down a flight can also be used to resolve problems and manage the most mundane or the most challenging everyday situations.

### Life As Meaningful

The transformations experienced go beyond behaviors and skills. Participants indicate that the changes are fundamental whereby life and the certainty of death have facilitated new meaning and values. For example, Dave spoke about fundamental values guiding life choices. Chase noted:

None of that shit (materialism) actually matters at the end of the day. … Just friendships, memories, experiences, you know… I think as extreme sports people we get to appreciate that a little bit more than the most… You just realize that life is finite, and there is an ending. We ARE all gonna die, that’s a fact. But it is all about how we LIVE, that actually matters. A lot of people don’t realize that they’re gonna die until they’re sixty something, and they’ve wasted their whole life on working in a cubicle. (Chase)

Here, Chase seems very clear that his desire to consciously choose how he lives his life is directly related to the realization that death is unavoidable and inevitable. Proximity flyers in this study report that not only is every proximity flight an active choice to embrace life but that proximity flying enhances the way they live their everyday life. Expanding on this Chase describes how everyday life is appreciated more deeply and enjoyed more. Chase puts this succinctly:

I will never be bored. I just like to go for a walk and I like to play music. I like to ride my bike and I like to climb. If I am by the ocean, I can walk up and down beaches all day. I go for swims, surfing… I will never be bored, cause I enjoy it too much. (Chase)

Chase perceived that proximity flying facilitated a love of life where the enjoyment he feels in life means that even seemingly mundane activities facilitate pleasure. Chase does not imply that he needs proximity flying to ensure that he is never bored but that meaning and joy can be found in less extreme activities such as walking by the ocean. For Chase, the new perspective on life gained through proximity flying enriches all aspects of his life. Fred describes this in terms of a realization that leads to a choice. Proximity flying acts as the facilitator to a realization that life is short and death is inevitable which in turn leads to a choice to use this as a reason to be sad or to live life and enjoy life.

I simply take a giant leap back and look at the reality: nothing is made to last, and we are all gonna die someday. You, me, my parents, my brother, your lover, everyone! So either you have a breakdown or you’re sad for the rest of your life, or you admit that reality and choose to enjoy, live, and love the rest of the tiny spark that is our short life in the infinite universe. (Fred)

In the quote above Fred shared a philosophy of life, which may have come to him through his involvement in proximity flying. His views are consistent with a meta-perspective, and thus give meaning to his involvement in extreme sport. This meta-perspective is consistent with the notions described by Wong (2008), who theorized that a positive acceptance of the reality of death can lead to a life lived with joy and meaning.

### Emergence of Selfhood

Extreme sport athletes often describe the experience of transformation as deep and profound, altering their sense of identity. For Adam flying is described as a central aspect of identity, a defining aspect of being free to be human:

The sport, flying, has taught me to know myself. I have come to know myself physically and mentally; my own patterns of reaction. I know myself all the way out to my fingertips, all these tiny movements. Or, I am aware of my own reactions when I am full of adrenaline and happiness, or full of fear. (Adam)

In the above quote Adam explains that proximity flying has facilitated a deep understanding of who he is in the world, physically and mentally. Adam does not suggest that proximity flying changes who he is and makes him into something new but that the experience of transformation is more of a realization of profound self-knowledge. Eric uses metaphor to describe this in depth:

It is like, it is who you are … What I think about when I see a bird that is caged, you know … A beautiful bird that is meant to fly, and he is caged. (Eric)

In the quotation above Eric draws on the metaphor of ‘caged bird.’ The ‘cage’ reflects a sense of limitation and has long been associated with terrestrial beings. The “bird” has long been linked to the potential ideals of freedom and inspiration often allied to flight. Eric uses the metaphor as a way of explaining that proximity flying is associated with the release from imposed normative constraints. Proximity flying represents freedom and capacity to explore beyond the realms associated with constrained beings. His metaphor encapsulates a fundamental aspect of who we are as humans, that is, the duality of limitations but also the potential for profound freedom.

## Discussion

Phenomenology seeks to give expression to lived-experience from a human science perspective. This requires that we “bracket our assumptions” as far as we are able and adopt methods consistent with psychology as a human endeavor. Furthermore, phenomenology assumes that humans have the potential for transcendence, in that we are able to gain a reflexive understanding of our own actions and understandings (Brooke, 2017). Participation in extreme sports is a prime example of human endeavor where individuals face their limitations and engage in an activity which challenges traditional notions of what it means to be human, that is, in the current context to move from artificially constrained beings to living more authentic lives. In this context, the findings from participants in this study point to the possibility of profound changes resulting for participating in such activities (Willig, 2008; Allman et al., 2009; Brymer and Oades, 2009). The motivation, attitudes, and skills realized through proximity flying, as also reflected in other extreme sport contexts, were not just context-specific but have been shown in related studies to exert positive influence in the everyday lives of athletes in a variety of social settings (Brymer and Schweitzer, 2017). The explication of the experience of participants in the current study illustrates the ways in which participants develop greater capabilities, grow as human beings, and transfer skills developed through learning to be effective in human flight, into their everyday lives.

For participants in this study, proximity flying has provided a medium for momentous realizations. As Willig (2008) reported extreme sports participation has the potential to facilitate deep meaningful discoveries about who we are and how we interact with our world. Rather than focusing on more traditional understandings based upon risk taking and the ‘extreme’ life, pushing boundaries and living on the edge; wingsuit pilots in this study were resolute that proximity flying affected them positively and facilitated the realization of the most profound aspects of self previously hidden from view (Holmbom, 2015, unpublished). The findings in this study are consistent with the notion that particular events may be instrumental in facilitating awareness of a deeper sense of self resulting in a profound and personal experience of transformation. These experiences are thus consistent with the transformative hypothesis previously posited (Brymer, 2013). Proximity flying affords fundamental changes in (1) a pilot’s sense of self and lived experience, (2) the way they make sense of the world, and (3) how they interact with the world.

Through participating in the extreme sport of proximity flying, participants in this study describe the development of a clearer perspective of personal values and understandings of life. Proximity flying, affording the experience of being close to the potential of one’s own death, facilitates profound self-awareness and a desire to live every day authentically. That is, a process of integration of the self and an experience of eudaimonic wellbeing. This notion is reflected in MMT (Wong, 2008) where the positive acceptance of death facilitates joy, wellbeing and meaning through a better understanding of personal wants, needs and desires.

While the findings from this study provide a glimpse into the possibilities realized through extreme sports and in particular proximity flying there are limitations that need to be recognized. A critique of the phenomenological approach is that the findings cannot be generalized to a broader population. Equally, phenomenology does not claim to link cause and effect. However, by linking the explication of lived experience to established theoretical frameworks phenomenology provides a deeper understanding of the lived experience of phenomena. Future research should develop the concepts explicated in this study to determine the capacity for extreme sport experiences to facilitate profound and life enhancing transformations more broadly.

Research into the extreme sport experience is still in its infancy and a great deal more needs to be investigated; future studies might explore exactly how extreme sport participation triggers personal transformations. Participants suggest that opportunities to experience vulnerability, connection, and the potential for death are important. Future studies may enhance our understanding of processes involved in facilitating transformation, the essential elements of the process and how these elements may be manipulated. This is, of course, not to suggest that the only way to achieve transformative experiences is through extreme sport, no doubt, there are many approaches which will lead to similar outcomes. Future studies might also investigate the link between extreme sport participation and human health and wellbeing as the transformations reported indicate that participants gain personal health and wellbeing, and social benefits from participation.

## Conclusion

In recent years, research into extreme sports has revealed that the experience is both more complex and more profoundly rewarding than previously assumed. For the most part, research has focused on motivations for participation and explicating the lived experience of the extreme sport activity itself. While some studies have pointed to the possibility that extreme sport participation might facilitate profound and positive transformations in the everyday lives of participants, the lived experience of this transformation has attracted less attention. This study undertook to describe the lived experience of the everyday lives of proximity pilots. Pilots describe enhanced self-awareness, and changes in values and identity that they perceive to result from their experience of human flight. Pilots describe experiences that reflect a sense of the phenomenological concept of authenticity. It may well be that certain events that have the potential to bring humans nearer to the reality of their finitude are also life-changing and enhancing experiences. The experience of transformation experienced by participants through extreme sports emerged as being connected to a realization of a deeper sense of self. This sense of self may well emerge in response to challenging the dictates of who participants ‘should’ be and how they ‘should’ lead life. We have argued that the extreme sport experience strips away the socio-cultural noise and allows an individual to hear an authentic own self. As such, the findings have implications for a broader population, as all humans have a potential to consistently realize their own authenticity through a range of modalities, which do not necessarily involve such extreme sports. However, extreme sports have the potential to provide a mirror of the ways in which human experience may be both challenging and also provide a greater understanding of human potential across multiple domains.

## Ethics Statement

This study was carried out in accordance to the requirements of Umeå University and the Swedish Research Council. All participants received written information concerning informed consent before agreeing to being interviewed.

## Author Contributions

All authors listed have made a substantial, direct and intellectual contribution to the work, and approved it for publication.

## Conflict of Interest Statement

The authors declare that the research was conducted in the absence of any commercial or financial relationships that could be construed as a potential conflict of interest.

## References

[B1] Allman T. L. Mittelstaedt R. D. Martin B. Goldenberg M. (2009). Exploring the motivations of BASE jumpers: extreme sport enthusiasts. *J. Sport Tour.* 14 229–247. 10.1080/14775080903453740

[B2] Bennett G. Henson R. Zhang J. (2003). Generation Y’s perceptions of the action sports industry segment. *J. Sport Manag.* 17 95–115. 10.1123/jsm.17.2.95

[B3] Berry M. Las Fargeas J. Blair K. B. (2010). Wind tunnel testing of a novel wingsuit design. *Proc. Eng.* 2 2735–2740. 10.1016/j.proeng.2010.04.059

[B4] Brooke R. (2017). *Psychology as a Human Science: Notes for Students.* Available at: http://rogerbrookephd.com/notes-on-psychology-as-a-human-science/ [accessed June 2 2017].

[B5] Brymer E. (2013). “Extreme sports as transformational tourism,” in *Transformational Tourism: Tourist Perspectives*, ed. Reisinger Y. (Wallingford: CAB International).

[B6] Brymer E. Downey G. Gray T. (2009). Extreme sports as a precursor to environmental sustainability. *J. Sport Tour.* 14 193–204. 10.1080/14775080902965223

[B7] Brymer E. Gray T. (2009). Dancing with nature: rhythm and harmony in extreme sport participation. *J. Advent. Educ. Outdoor Learn.* 9 135–149. 10.1080/14729670903116912

[B8] Brymer E. Oades L. G. (2009). Extreme sports: a positive transformation in courage and humility. *J. Humanist. Psychol.* 49 114–126. 10.1177/0022167808326199

[B9] Brymer E. Schweitzer R. (2013). Extreme sports are good for your health: a phenomenological understanding of fear and anxiety in extreme sport. *J. Health Psychol.* 18 477–487. 10.1177/1359105312446770 22689592

[B10] Brymer E. Schweitzer R. (2014). The search for freedom in extreme sports: a phenomenological exploration. *Psychol. Sport Exerc.* 14 865–873. 10.1016/j.psychsport.2013.07.004

[B11] Brymer E. Schweitzer R. D. (2017). *Phenomenology and Extreme Sports.* Abingdon: Routledge Press.

[B12] Celsi R. L. Rose R. L. Leigh T. W. (1993). An exploration of high-risk leisure consumption through skydiving. *J. Consum. Res.* 20 1–23. 10.1086/209330

[B13] Elmes M. Barry D. (1999). Deliverance, denial, and the Death Zone: a study of narcissism and regression in the May 1996 Everest climbing disaster. *J. Appl. Behav. Sci.* 35 163–187 10.1177/0021886399352003

[B14] Emmons R. (1999). *The Psychology of Ultimate Concerns: Motivation and Spirituality in Personality.* New York, NY: Guildford Press.

[B15] Franques P. Auriacombe M. Piquemal E. Verger M. Brisseau-Gimenez S. Grabot D. (2003). Sensation seeking as a common factor in opioid dependent subjects and high risk sport practicing subjects. A cross sectional study. *Drug Alcohol Depend.* 69 121–126. 10.1016/S0376-8716(02)00309-5 12609693

[B16] Hoggan C. D. (2016). Transformative learning as a metatheory: definition, criteria, and typology. *Adult Educ. Q.* 66 57–75. 10.1177/0741713615611216

[B17] Kerr J. H. Houge Mackenzie S. (2012). Multiple motives for participating in adventure sports. *Psychol. Sport Exerc.* 13 649–657. 10.1016/j.psychsport.2012.04.002

[B18] Larkin M. Griffiths M. D. (2004). Dangerous sports and recreational drug-use: rationalizing and contextualizing risk. *J. Community Appl. Soc. Psychol.* 14 215–232. 10.1002/casp.770

[B19] Le Breton D. (2000). Playing symbolically with death in extreme sports. *Body Soc.* 6 1–11. 10.1177/1357034X00006001001

[B20] May R. (1983). *The Discovery of Being: Writings in Existential Psychology.* New York City, NY: W.W. Norton & Company.

[B21] Mei-Dan O. Monasterio E. Carmont M. Westman A. (2013). Fatalities in wingsuit BASE jumping. *Wilderness Environ. Med.* 24 321–327. 10.1016/j.wem.2013.06.010 24238216

[B22] Monasterio E. (2007). *The Risks of Adventure Sports/People. The Alpinist 19 November.* Available at: http://www.alpinist.com/doc/web07f/rb-erik-monasterio-mountaineering-medicine [accessed June 11 2008].

[B23] Monasterio E. Alamri Y. A. Mei-Dan O. (2014). Personality characteristics in a population of mountain climbers. *Wilderness Environ. Med.* 25 214–219. 10.1016/j.wem.2013.12.028 24703096

[B24] Norton D. L. (1976). *Personal Destinies.* Princeton, NJ: Princeton University Press.

[B25] Pain M. T. Pain M. A. (2005). Essay: risk taking in sport. *Lancet* 366 S33–S34. 10.1016/S0140-6736(05)67838-5 16360743

[B26] Park J. (2006). *Our Existential Predicament: Loneliness, Depression, Anxiety and Death.* Minneapolis, MN: Existential Books.

[B27] Rogers C. R. (1961). *On becoming a Person.* Boston, MA: Houghton Mifflin.

[B28] Self D. Henry E. Findley C. Reilly E. (2007). Thrill seeking: the type T personality and extreme sports. *Int. J. Sport Manag. Mark.* 2 175–190. 10.1504/IJSMM.2007.011397

[B29] Simpson D. Post P. G. Tashman L. S. (2014). Adventure racing: the experiences of participants in the Everglades Challenge. *J. Humanist. Psychol.* 54 113–128. 10.1177/0022167813482188

[B30] Slanger E. Rudestam K. E. (1997). Motivation and disinhibition in high risk sports: sensation seeking and self-efficacy. *J. Res. Pers.* 31 355–374. 10.1006/jrpe.1997.2193

[B31] Smith J. A. Flowers P. Larkin M. (2009). *Interpretative Phenomenological Analysis: Theory, Method and Research.* London: Sage.

[B32] Westman A. Rosén M. Berggren P. Björnstig U. (2008). Parachuting from fixed objects: descriptive study of 106 fatal events in BASE jumping 1981–2006. *Br. J. Sports Med.* 42 431–436. 10.1136/bjsm.2008.046565 18523039

[B33] Wiersma L. D. (2014). A phenomenological investigation of the psychology of big-wave surfing at Maverick’s. *Sport Psychol.* 28 151–163. 10.1123/tsp.2013-0001

[B34] Willig C. (2008). A phenomenological investigation of the experience of taking part in “extreme sports”. *J. Health Psychol.* 13 690–702. 10.1177/1359105307082459 18519442

[B35] Willig C. (2013). *Introducing Qualitative Research in Psychology*, 3. Edn. Berkshire: Open University Press.

[B36] Wong P. T. P. (1998). “Spirituality, meaning and successful aging,” in *The Human Quest for Meaning: A Handbook of Psychological Research and Clinical Applications*, eds Wong P. Fry P. (Mahwah, NJ: Lawrence Erlbaum), 359–394.

[B37] Wong P. T. P. (2000). “Meaning in life and meaning in death in successful aging,” in *Death Attitudes and the Older Adults: Theories, Concepts and Applications. Bruner*, ed. Toner A. (Philadelphia, PA: Routledge), 23–35.

[B38] Wong P. T. P. (2008). “Meaning management theory and death acceptance,” in *Existential and Spiritual Issues in Death Attitudes*, eds Tomer A. Eliason G. T. Wong P. T. P. (Hove: Psychology Press), 65–90.

[B39] Wong P. T. P. Tomer A. (2011). Beyond terror and denial: the positive psychology of death acceptance. *Death Stud.* 35 99–106. 10.1080/07481187.2011.535377 24501830

[B40] Woodman T. Hardy L. Barlow M. Le Scanff C. (2010). Motives for participation in prolonged engagement high-risk sports: an agentic emotion regulation perspective. *Psychol. Sport Exerc.* 11 345–352. 10.1016/j.psychsport.2010.04.002

[B41] Zimmerman M. (1986). *Eclipse of the Self: the Development of Heidegger’s Concept of Authenticity.* Athens, OH: Ohio University Press.

[B42] Zuckerman M. (1994). *Behavioral Expressions and Biosocial Bases of Sensation Seeking.* Cambridge: Cambridge University Press.

